# Gender and intersecting vulnerabilities on the mental health unit: Rethinking the dilemma

**DOI:** 10.3389/fpsyt.2022.940130

**Published:** 2022-09-23

**Authors:** Elizabeth Kathleen Morton, Sarah K. McKenzie, Amy Cooper, Susanna Every-Palmer, Gabrielle Lisa Simone Jenkin

**Affiliations:** Department of Psychological Medicine, University of Otago, Wellington, New Zealand

**Keywords:** gender, mental health, service users, acute, facility, architecture, vulnerabilities

## Abstract

**Background:**

Gender is routinely pitched as a key determinant of vulnerability for staff and residents on acute mental health inpatient units. Since the 1960's mixed gender units have become more prominent in Western health systems, yet questions remain around the configuration of these units, including how to ensure emotional and physical safety of those living and working in them.

**Methods:**

This paper draws on a large study of the lived experiences of 42 staff and 43 service users from different acute mental health units in New Zealand. We conducted thematic analysis of interview data from four units with diverse architectural layouts to identify key themes central to decisions around gender and spatial design.

**Results:**

Key themes emerged around gender-related trauma histories, safety perceptions and vulnerabilities, accommodation of gender-diverse and non-binary mental health service users, and gender-specific needs and differences. A further theme, of *it goes beyond gender* emphasized that there are many other non-gender attributes that influence vulnerability on the unit.

**Conclusions:**

While findings emphasize the need for safe places for vulnerable people, trauma-informed care, access to staff who “understand,” and recreation that is meaningful to the individual, we question if the dilemma of gender-separation vs. gender-mixing is an outmoded design consideration. Instead, we argue that a flexible, person-centered approach to provision of care, which values autonomy, privacy, and safety as defined by each service user, and that promotes choice-making, obviates a model where gender accommodations are fore. We found that a gender-exclusive narrative of vulnerability understates the role of other identifiers in dynamics of risk and vulnerability, including age, physicality, past violence, trauma history, mental unwellness, and substance use. We conclude gender need not be a central factor in decisions around design of prospective built unit environments or in occupational and clinical decisions. Instead, we suggest flexible spatial layouts that accommodate multiple vulnerabilities.

## Introduction

Until the 1960's, psychiatric inpatient facilities in New Zealand were segregated by sex (1). The “phasing in” of gender-integrated units coincided with a move toward deinstitutionalisation, initiated by a decision to halt the planning and construction of further institutional accommodation (2). The shift to mixed-gender units emanated from a more transitory model of mental health inpatient care internationally, with greater focus on community supports and the mirroring of “real world” conditions (3–5). However, normalization of gender mixing could arguably mirror and augment problems experienced by women in the world outside the unit, which include a disproportionate experience of male-instigated violence (6).

A considerable body of research exists on the gendered aspects of life in adult acute mental health facilities (3, 4, 7–13). This literature is supplemented by research in specialized forensic (14–18), general hospital (19–22), and outpatient mental health (23–26) settings. Many papers examine the experiences of women (6, 7, 11, 12, 15, 18, 27–46), while the perspectives of men are notably absent in much of the qualitative research. This can be partially explained in terms of a phenomenon of “concealment” whereby men are more likely to mask indicators of vulnerability, ultimately lending to outsiders being “blinded” to male trauma experiences (47). Men are, in this way, often excluded from status as a vulnerable population.

Literature specific to service user and staff perspectives on gender separation and mixing in acute mental health settings exists, but there are caveats to the depth, translational capacity and scope of some of this research. Most studies explored attitudes within contemporaneous units of different setups (12, 48–50). Various qualitative and quantitative research have sought to understand attitudes toward mixed-gender and gender-separated design, or to get a sense of the behavioral phenomena that manifest within these environment types (8). Qualitative studies have compared the views and experiences of staff and service users across mixed-gender and gender-segregated unit settings. Two studies from the United Kingdom examined the impact of changing psychiatric units from mixed-gender to gender-segregated (13, 51). The first, a unit re-configured from a mixed-gender to a men only unit, showed an increase in staff perceptions of aggression (13), while the latter found the conversion of two mixed units into two segregated units resulted in a ‘calmer' men's unit, but more disruption on the women's unit (51). Another looked at service user perspectives over a change from gender-segregated to mixed-gender, finding that non-gender factors, such as younger age and co-morbid substance use issues, positively correlated with a preference for gender mixing (52). Studies have conflicting findings on which model is preferred, with some concluding service users preferred gender-segregated accommodation (53), and others favoring gender mixing (52). A majority of studies reviewed found more female service-users preferred single-gender inpatient facilities than their male counterparts (48, 52, 54).

However, there are limitations to current understandings of gender and issues of gender-separation or mixing in the acute mental health setting. A proclivity for service users to endorse the therapeutic milieu to which they were admitted–and hence familiar with–was sometimes shown (3, 41, 52), making conclusions about preference and optimum design difficult. Further, studies of staff experiences and preferences are mostly limited to those of nursing staff. The positioning of separation vs. mixing as a base for qualitative inquiry has been criticized for creating a misnomer of simplicity in the face of a problem that is complex, textural and nuanced (55). Some authors recommend future “in-depth research” around the full phenomenology of gendered experiences on the unit, as well as discussion around provision of “women only spaces” (3, 55, 56). Accommodation of gender differences and needs, adds complexity to development and delivery of models of care in acute mental health facilities characterized by a diverse service user population. Literature shows that a person-centered, identity-relevant, approach in healthcare can benefit treatment engagement, treatment satisfaction, and treatment outcomes (57, 58).

Experiences of staff and residents in the mental health unit are frequently described in terms of being traumatic (59, 60). A history of gender-related trauma that precedes admission has been shown to amplify feelings of unsafeness in mixed-gender inpatient units (32). Issues of physical safety, especially for women on the unit, are evidenced by reports of harassment and violence (27, 61, 62). Gender based violence, in particular violence toward women, is often pitched as a key incarnation of trauma and abuse on the unit and in the life histories of service users preceding admission (27, 63, 64). However, the positioning of gender based violence as meaningful to discussions around unit violence is challenged by the understanding that a heteronormative framing of male-to-female violence underplays the lived experiences of many men (65) and “rainbow” or LGBTQIA+ service users, including those Māori-defined identities of Takatāpui (same sex oriented persons) and Irawhiti (transgender and gender diverse persons) (66). Childhood abuse, and more narrowly childhood sexual abuse, is a reality for many men (67), especially men who have experience of inpatient services (68). Given configurations of abuse can operate between people of the same gender, and that men and women can sit on both sides of a perpetrator/victim formulation or vacillate between roles, the pitching of unit violence as “gender based” is theoretically weak. In this paper we use “sexual violence” and “sexual harassment” where more relevant, and use gender based violence only when gender is positioned as the salient factor. In doing so, we recognize that women are reported victims of sexual abuse significantly more often than men (6).

There is increasing interest in the experience and accommodation of gender-diverse service users within the mental health system (24, 26, 69–72). A review of literature suggests the lexicon of gender has altered in recent years to reflect a less binary formulation, in keeping with an enterprise to work with gender-inclusive language and diverse identities (73). Early literature held biological “sex” as central to discussion of service users within mental health units, whereas “gender” is more prominent in the current discourse. This paper uses “mixed-gender” and “gender separation,” rather than “mixed-sex” and “single-sex” in discussions relating to mental health facility layout and organization.

There is little research around the New Zealand experience, with the only substantive literature identified being a Master's thesis (1). From a feminist perspective, Hewitt analyzed qualitative data collected from seven women and one man who had spent time on psychiatric units, four as nurses and three as service users. This thesis provided a historically contextualized, qualitative exploration of gendered experiences and attitudes around gender-mixing in the local setting, concluding that a notion of “normalization” used to justify the mixing of genders can place women at emotional and physical risk in the “abnormal” context of an acute mental health setting. However, the small sample and the fact this research is now 20 years old constrains generalisability of findings.

This paper aimed to answer the research question: “What are gendered experiences on the acute mental health unit, and what are the spatial implications for unit design and model of care?”. Using data from a large multisite qualitative project exploring New Zealand staff and service user experiences of acute mental health facility design (74), this study focuses specifically on experiences in relation to gender to determine its salience with regards to spatial design and model of care.

## Methods

This paper reports on a qualitative analysis of interview data collected during a 3-year study to understand the built therapeutic and social environment of New Zealand's adult acute psychiatric inpatient facilities. As part of this we were interested in the socio-political organization of space on the units. In the current analysis, we focused on the gendered experiences of service users and staff in four mental health inpatient units. Gendered experiences are those by which the gender of interviewees, or their perceptions of gender as it impacted other parties, is relevant to their experience on the unit, and to perceptions of the unit environment and social milieu.

### Ethics, consultation, and locality approvals

As part of the initial development of the primary study, consultation with Otago Ngāi Tahu Research Consultation Committee was initiated, as per University of Otago criteria, to involve indigenous Māori in a review of the research proposal. The ethics application (17/CEN/94) was reviewed and approved by the Central Health and Disability Ethics Committee. Locality consent was granted by the four participating District Health Boards. The study protocol is available in the Australian and NZ Clinical Trials Registry (ACTRN12617001469303): http://www.ANZCTR.org.au/ACTRN12617001469303.aspx. The study protocol assured the participating hospitals that they would net be identifiable in the research results.

### Data collection

Data collection involved multiple site visits to inpatient units across New Zealand between 2017 and 2019 by GJ, a social scientist, to conduct interviews and collect other data on unit design. Four inpatient facilities, ascribed here as Unit A, B, C and D, were selected out of a sample frame of all 20 publicly funded mental health units in New Zealand. Case facilities were selected for diversity, in terms of building age, condition, design and location. The first four units prioritized for inclusion on this basis agreed to participate. All units were publicly funded, mixed gender inpatient units, which admitted acutely unwell adults between the age of 18 and 65 for short term care.

#### Participant invitation

Posters and study information sheets were displayed in the main circulation areas of the units and in the staff room prior to and during the study, and the principal investigator worked with the management and senior staff to promote the study and recruit participants. This required frequent daily visits to the units over 7–10 days, with repeat visits over a 2-month window for some units where recruitment was more challenging.

#### Recruitment of staff

Recruitment involved invitation of staff across the range of disciplines on the unit, with the aim of including several nurses, and ideally one of each from the other professions on the unit; care assistants, social workers, occupational therapists, psychiatrists, cultural advisors and pharmacists. Staff within the priority occupations for interview then self-selected to be interviewed and were interviewed in their work time. Much of this was organized verbally by conversations between the lead investigators and the staff involved.

#### Recruitment of service-users

As per ethics approval, a list of service users who were assessed by their lead clinician as competent to consent, well-enough for the interview, and potentially interested in participating, was provided to the lead investigator, GJ. With the help of senior staff on the ward, the principal investigator went onsite to find the listed individuals and arrange interviews with those who agreed to participate in the study, interviewing service users who provided written consent. On a number of occasions some service users changed their mind about participating in the research by the time the interview could be arranged: several decided they no longer wanted to participate, so the recruitment period was extended until enough participants had been interviewed.

Due to resource constraints a decision was made to cap the number of interviews on each ward to 20, allowing 10 with each of the staff and service user groups, although more were interviewed in reality.

### Interviews

Interviews lasted 30–90 min, were audio recorded and transcribed verbatim. All but 10 took place in a private room on the unit in a face-to-face setup, with the remainder conducted by phone.

### Analysis

Interview transcripts were read thoroughly by three authors (EM, AC, GJ), who then met with SM and SEP to discuss the data. Relevant extracts of the data were then prioritized for further analysis. The research agenda was to an extent *a priori*, insofar as exploration around gender on the unit was sought. A broadly deductive approach enabled discovery of gender-related themes, while answering a defined research question. This said, our approach assumed a dynamic relationship between the findings and the research questions. We employed thematic analysis, as described by Braun and Clarke (75), which involves pattern-finding to represent “meaningful groups” of data (75), with stipulation of emergent themes based on their “keyness” (76). Sections of staff and service user transcripts relating to gender as it impacts unit life, as well as attitudes toward gender and gender-related issues, were coded line by line by the first author (EM). These were subsequently assigned key and sub themes, and these were discussed and agreed upon by the five contributing researchers. These individuals, with diverse backgrounds in social science, public health, medical scholarship and mental health inpatient service-user experience, met over several virtual workshop sessions to review, dynamically revise, and agree on key themes. In an iterative process, the research direction was honed to best capture the “stories” within the data.

Data on the spatial organization of the units were collected and documented through photographs and architectural plans of unit layouts. Interview transcripts, which were anonymised and are reported here in a codified form, indicate: the ward (A, B, C, or D), their position as mental health service user (MH) or staff (S), their gender (W or M, T =Transgender), their Ethnicity (indigenous Māori or non-Māori), and their interview number. For example, A_MH_M_NM_4 = Unit A, Mental health service user, Male, non- Māori, participant 4 (of service user group in Unit A).

## Results

### Participant and unit characteristics (layouts and design)

Table 1 shows the participant characteristics by ward, and the unit layouts and relevant design features for each of the four units.

**Table 1:** Unit and participant characteristics.

	**Case A**	**Case B**	**Case C**	**Case D (New)**
**Participants**				
Service users–men	4	6	5	5
Service users–women	5	5	7	5
Transgender	1	0	0	0
*Total service users*	10	11	12	10
Staff men	5	6	4	0
Staff women	4	7	7	9
*Total staff*	9	13	11	9
Total	19	24	23	19
**Unit Characteristics**				
Beds	22	64	21	32 (first build)
Site situation	On hospital grounds	Not attached to hospital on own park like campus	On hospital grounds	On hospital grounds
Bedrooms	All single	All single	All single	All single
Gender separated bedrooms/or wings	Yes–some gender separation by corridor with attempts to locate women in the bedrooms nearest the nurses' station	No	Yes. Gender separated wings of bedrooms with 2 gender neutral/family bedrooms at top end of male and female corridor close to the nurses' station, which was used for vulnerable persons.	No
Bathrooms	Unisex (a couple of bedrooms have own bathroom)	Some unisex, some female, some male (In reality people used which ever bathroom was free)	Gender separated bathroom on each wing, and ensuites for the 2 gender neutral bedrooms	All bedrooms have own bathroom
Can bedroom door be locked by service user	No	No	No	Yes
Bedroom door window curtains can be closed from inside by service user	No	Some have curtains on the inside	All have curtains on the inside	Yes, by a blind inside the double glazing that can be controlled from the outside and inside
Lounges	One unisex lounge	At least one woman only lounge the rest unisex	Has separate women's lounge on female corridor, and a small space for men at the end of the men's corridor (but not a proper lounge)	Have only unisex lounges. But has a flexi unit which is used for up to three vulnerable service users (with own courtyard and lounge)
Number of courtyards	1	4	1	2
Unit courtyards	Smoking in courtyard	Smoking in courtyard	Smoking in courtyard	Smoke-free courtyards

As shown in Table 1, a total of 85 interviews were conducted over the four mental health units (43 service users and 42 staff).

The relevant spatial aspects of the units' designs in terms of gender are shown in Table 1. These reveal diverse gender spatial arrangements of rooms, corridors and bedrooms. In terms of the age of the units, Unit A, C and D were much older, and Unit D was brand new, but only halfway through the build (two of the four sections were complete).

### Thematic findings

Five central themes, common to both staff and service users, were identified as relevant to the exploration of gendered aspects of social life on the unit as influenced by the spatial layout:

1. Gender-related trauma preceding admission.
2. Gender-related perceptions of safety and vulnerability on the unit.
3. The accommodation of gender-diverse and non-binary service users.
4. Gender-specific differences and needs.
5. Non-gender attributes that may influence risk and vulnerability on the unit.

**1. Gender-related trauma of service users preceding admission** to the mental health unit was consistently reported or conjectured by staff and service users. This was notable across all four units, and reported most often by women service users, and by staff. Many service users described gender-related trauma as both observation of others, and as self-observation.

Service users often interpreted their mental distress, and that of their peers, through a traumagenic lens.

The most common pre-admission traumas discussed by participants related to violence toward women, inflicted by men. As well as preventing sexual violence on the unit, a repeatedly voiced reason for gender separation by staff was a recognition of trauma histories by women and the importance of ensuring their emotional safety:

*Many of the women that come through, have had situations of abuse in their lives. And surely to God we can keep them separate from the guys*. (A_S_M_NM_8)

*And we have also had a vulnerable woman that had been raped and things like that that don't like being around men. So, it's easier to keep the two apart*. (C_S_W_NM_1)

Some women were keenly aware of the potential trauma histories of other women. One woman framed the trauma of a fellow female service user's experience of rape as a consequence of women's vulnerability in a patriarchal society:

*She's gone through a significant amount of pain. And what it is, is pain that being a female in a male-dominated world, and not having people that understand the implications of rape*. (B_MH_W_M_1)

Some women service users described post-traumatic difficulties co-habiting with men on the unit, which contributed to feelings of discomfort and lack of safety:

*if say you're a female who's been through trauma, the last thing you want to do is be in a closed room with men*. (A_MH_W_M_2)

However, while male to female sexual violence is undoubtedly more common, men also talked about vulnerability to trauma–as one male service user reflected on his own trauma preceding admission:

*I'd like to say - hey you know things happen, you know. Things have happened in my life, like I've had all this trauma deep, deep, deep, deep, deep down* (A_MH_M_NM_4).

Staff were cognisant of the inherent difficulties of accommodating service users with past experiences of gendered violence (violence by the opposite gender) in a mixed unit setting:

*Many have been traumatized by the opposite gender, and we're forcing them right into each other's faces*. (A_S_W_NM_4)

This observation was notably non-specific as to the gender dynamic at play. Men and women could assume roles as victim or perpetrator. Provisions to better attend to the needs of individuals with trauma histories were noted by a number of staff. These included gender separation, surveillance, alternative restraint practices, and talk therapies. Both women service users and staff recognized the possibility of re-traumatisation for individuals by being in the unit environment. One staff member, on being asked why a unit had attempted to separate females from males, explained how the lack of space on the unit and mixing of genders exacerbated the traumagenic effects of the environment:

*forcing patients like this, all in crisis, into a little wee TV room on top of each other, males and females together, where many have been traumatized by the opposite gender, and we're forcing them right into each other's faces. And I think their ability to settle and resolve, to settle their mental state, is reduced and takes a lot longer, because of the way that our ward's set up. It actually re-traumatizes them*. (A_S_W_NM_4).

One way of reducing this re-traumatisation and providing for the needs of trauma-impacted individuals is the use of ‘flexi' spaces. These featured in Unit D with a mixed gender layout, where a separate pod or bubble of 2–3 bedrooms (with their own courtyard, lounge and bathroom, and own staff), are located in between the open and closed wards (with doors that can be opened either the high needs ward or the open ward by staff as needed). This effectively accommodates a small group of service users who are in a vulnerable place or stage of their wellbeing.

*A lot of our female clients have had trauma in the past…, and so we do try to take that on board when we can. So we do have areas called flexi areas where they can be nursed away, not necessarily in female-only spaces, but they can be nursed away from the [male participants on the] ward*. (D_S_W_M_5)

Some facilities provided women's lounges, for example, Unit B. However, this differential treatment did not go unnoticed by some male service users who felt this was unfair:

*The only thing that annoys me, is the fact that there's a women's lounge and not a man's lounge. The women have a place to go to get away from the men, but there's no place where the men can get away from the women*. (B_MH_M_NM_9)

An alternative design strategy was suggested by one staff member, with common areas set up with ‘little nooks and crannies, that people can sit and chill' (C_S_W_NM_1). While not explicitly relevant to gender needs on the unit, satisfying service users' need for space and choice-making may attenuate feelings of vulnerability and unsafeness. Staff and service users were aware of lack of space in the communal areas of some of our case units, as well as seating limitations:

*At the moment there's not enough tables and chairs for patients* (C_S_W_NM_1).

One staff member suggested the policy goal in acute mental health care settings of using human restraint (hands on immobilization of the service user) instead of seclusion, was particularly problematic and traumatizing for those already suffering from histories of abuse:

*They're trying to push zero seclusion by 2025 and have only physical restraints. So what about the people that had been raped and abused? They don't want hands on, they'd rather a room to themselves*. (D_S_W_NM_2)

Some staff were cognisant of the fact that gender of the person/s doing the restraining is also likely to be a factor of significance to the person restrained and their specific trauma history, and that this could retraumatise them. The staff member (D_S_W_NM_2) highlighted the need to recognize within-group differences in preferred treatment options.

**2. Gender-related perceptions of safety and vulnerability** on the unit were described by both service users and staff. Often, these perceptions were used to justify the need for gender separation on the unit. Issues of safety concerned both the welfare of service users and staff. Recognition of safety issues had implications for the recruitment and rostering of staff, utilization of occupational or diversional activities to mitigate agitation and violence, and the use of unit design to facilitate safe spaces for service users to commune and to sleep.

Women service users were most likely to voice concerns about unit safety. While it was not always explicit that safety concerns and incidents of unit violence followed a male-on-female configuration, women expressed concern for other women.

*I wouldn't wish that upon anyone … regarding the safety to think that it was okay to go in there [in the unit], especially if it was my granddaughters or my daughter*. (C_MH_W_NM_5)

Again, feelings of unsafeness were sometimes linked to the mixed gender unit layout. Staff and service reported that at times men entered women's bedrooms. A service user on the gender mixed Unit D, commented on the lack of gender separation of bedroom areas:

*Right next door, there's a guy in there, yeah. It's just not safe*. (D_MH_W_M_7)

Staff described some of the men on the unit in pejorative terms, as “predatory,” “philandering” and “violent.” The potential for danger where these men are in mixed gender units was noted by a staff member:

*We're managing aggressive, aggro males, that have used drugs, and have no belief that they're unwell, with your grandmother*. (B_S_M_NM_3)

In this, gender was just one of multiple vulnerabilities and risk factors identified by staff perceived to affect safety.

Sometimes male-on-female violence was determined to be a result of a dynamic of unwellness, while some staff saw elements of opportunistic intent. The gender mix of staff on shift was seen as relevant to the incidence of premeditated assaults in some cases:

*When you saw this guy come along and he knew he's gonna be violent and you could see the guy looking around counting, doing a headcount, and thinking ‘is it worth me being assaulted? No, too many men' … And if you've got mainly female nurses, that's just how they get their needs met*. (C_S_W_NM_11)

Notably, some staff viewed service user violence on the unit as enacted by ‘any gender' (B_S_M_NM_6). Further, a staff member suggested that *vulnerability* to violence is not gender or sexuality specific:

*It doesn't make any difference these days, does it, as far as sexual preferences and things, so anyone could be at risk gender-wise*. (B_S_W_NM_8)

This staff member appears to describe the increasing understanding that non-heteronormative identities and sexual orientations indicate vulnerability that goes beyond a simple gender formulation. Violence in New Zealand impacts lesbian, gay and bisexual (77), as well as transgender or non-binary (78) people to a disproportionate degree.

A staff member also commented that in their view, some unwell service users “cannot distinguish” between men and women, when lashing out in response to psychosis-related threats “They're just danger, danger” (D_S_W_NM_1).

Staff accounts of gendered safety issues on the unit sometimes invoked the narrative that women were more vulnerable when unwell, and that sexual activity and sometimes assault of women could be a result of illness-related hypersexuality, specifically as a symptom of mania presentations. In these accounts, women were more often seen as the vulnerable parties in sexual assault, while men were more often seen as perpetrators, though disinhibition and diminished comprehension of events were speculated:

*People become sexually disinhibited as well: I guess maybe someone who's manic, or if it's a bipolar disorder, or being psychotic. People can do things that they wouldn't necessarily do when they're well*. (B_S_W_NM_9)

Sexual relationships between service users on the ward were talked about as a common occurrence that raised important issues of sexual safety. A number of staff said they discouraged romantic and sexual relationships between service users on the unit because of the nature of the environment and the vulnerability of those in it.

*We don't encourage it. Because I mean ... You're meeting in a place that you know... you're both put here. You didn't both decide to come here to meet up*. (B_S_M_NM_4)

Other staff expressed concerns about how they could support people's autonomy while also keeping them safe:

*So I'm thinking about the safety of both [parties], but its the individuals'choices at the end of the day*. (A_S_M_NM_9)

Staff worried that service users might not be well-enough to give informed consent, that a power imbalance might exist between those “at different points in their recovery,” and that unwell service users might make sexual decisions they later regretted.

*A lot of these guys are mentally unwell ... they're vulnerable.... some of them don't really know what they're doing*. (A_S_W_M_1)

Service users did not share the staff view on forbidden romance on the unit, explaining that relationships established on the ward could be meaningful and long term.

*I met a girl here in 2014. We dated for a year and a half up until March or May 2015*. (A_MH_M_NM_5)

But accounts also provided some hints of impulsivity around relationship decisions, as can happen outside the mental health unit.

*And this time last week I met a woman here and we're actually planning to get married* (A_MH_M_NM_5)

Staff expressed discomfort and uncertainty around how best to manage these romantic relationship situations. They described a culture of not openly discussing the sexual and relational needs of service users. There was the sense that it was a complex issue, one that was often dealt with by pretending it did not exist, but this led to additional risks.

*There are no signs up about um, you know, how you can keep yourself safe [sexually]. And we've got a lot of vulnerable people here* (D_S_W_NM_3)

Some staff raised the matter of sexual frustration amongst some of the service users in the unit:

‘*If a male or even a female … is staying in a ward for a long long time … and we talked about their needs. What about their sexual needs?'* (D_S_W_M_8)

When this issue was raised with management its was however ignored:

*Nobody answered me of course, and I didn't expect an answer* (D_S_W_M_8)

**3. Accommodation of gender-diverse and non-binary service users**

Understanding the needs and accommodations of gender-diverse and non-binary service users is also relevant to attitudes about the separation or mixing of genders on the unit. Discussion related to these issues was found in staff interviews, and in comments from a transgender service user who identified as a woman:

*it could be more welcoming and better suited, especially for the gay and lesbian [and] trans community as well*. (A_MH_T_NM_1)

When asked about her attitude toward gender separation of bedrooms on the unit, she expressed reservations about full gender separation, explaining that it need not be “necessarily, like, segregated,” but responded positively to the notion of some separate spaces for people (like her) who identify as women:

*It would be nice just to have a separate area where people like myself could just chill out and connect in some way and support each other* (A_MH_T_NM_1)

The service user account suggested that gender-diverse populations are sometimes initially misgendered or allocated bedrooms in corridors with the non-identified gender:

*I was with nearly all men, down the corridor, and then I was moved into, where it's mostly women, where I identify as* (A_MH_T_NM_1)

Staff had varying levels of understanding of the accommodation needs of gender diverse service users. Some staff members said that the service user should elect the type of unit “where they would prefer to be.” Others considered this to be a decision for the “government and ministries,” or spoke of the thorniness of gender logistics as “such a hot topic these days.”

The provision of unisex toilets was suggested as a good design strategy by some staff:

*If they could be unisex, I think that's a bit better for people who have concerns around their gender identity things*. (B_S_M_NM_6)

The provision of “flexi wings” (Unit D) and “swing beds” (Unit C) was stipulated as useful in accommodating transgender individuals:

*we've got rooms in between them. And what I like about those rooms, because I also work with transgender clients … that's really, really good for that*. (C_S_M_NM_5)

*I think it's important if they have those in-between ones for gender* (C_S_W_NM_11)

Potential controversies about the bedroom allocation of gender-diverse service users was related by one staff member. This pertained to a previous unit set-up:

*I can remember many years ago… an issue where a transgender client wanted to sleep in what was a female dormitory. They identified as female. The people who most vociferously opposed that were the female clients*. (A_S_M_E_5)

Failure of the unit layout to satisfactorily provide safe and suitable accommodation of gender-diverse service users was described by some staff. Limiting factors mentioned included the financing of a unisex corridor–“we'd have to have three things set up” (these being a male, female, and unisex wing), resistance within the cis-gender service user population, and the idea that different transgender service users had different requirements–“one size doesn't fit all.” But the most common sentiment was one of recognition of need:

*We need to accommodate this. But we don't, we can't possibly accommodate them in the situation we've got. We need to be able to*. (A_S_M_NM_8)

**4. Gender-specific differences and needs**

Gender specific differences in the nutritional, recreational, hygiene, and staffing needs of both men and women service users were reported by staff and service users across the four units.

In terms of nutrition, meal portion sizes and the unavailability of snacks and facilities to prepare food in-between meals were criticized as inadequate by staff and service users at several Units. Some staff and service users highlighted portion size as a need expressly relevant to men:

*A lot of them say the portions aren't as big over here. But these guys, you get a lot of these guys over here, especially the boys, they're big eaters. They're big strapping guys* (A_S_W_M_1)

Similarly, available physical activity within the units was criticized by staff and service users as insufficient and lacking in relevance to the needs and interests of men, sometimes young men in particular:

*If some of these guys could just run up and down, up and down, up and down, or have a full basketball court or something, I think that would really, really help*. (B_S_W_NM_8)

*I think physical exercise would benefit especially our young men… I think they need something physical because we're talking young boys here. We're talking about the young men … You know, coloring in pictures ain't it*. (D_S_W_M_8)

Provision of punching bags and basketball hoops and fixed exercise equipment were suggested by some men service users and staff in some units. Unit A had a basketball hoop, though this was broken at the time of interviews. Men service users on Unit D had indicated their wish for more male-relevant exercise options in a signed petition sent to the management (which was and reportedly disregarded).

Occupational activities offered on the units were often viewed as skewed toward women's interests, and seen as too passive, infantile, or culturally inappropriate for men.

*There's ten of us males, in this ward… Those coloring in and mindfulness stuff, that's what my baby does*. (D_S_W_NM_9)

*Drawing therapy is not appropriate for them. We had a kaumātua [Respected Māori elder] years ago [based on the unit]… and he would do carving and the boys just loved that… And, you know, there's none of him around*. (C_S_W_NM_2)

In relation to personal hygiene, many women service users on units where unisex bathrooms were prevalent expressed clear differences in hygiene needs, in support of gender-segregated or ensuite bathrooms. These comments were especially present in the interviews from units with very limited number of ensuite bathrooms (units A and B) compared with Unit D where all bedrooms had ensuites.

*Men and women use bathrooms for different reasons … Men jack off, and get cum everywhere …. so you've got the thing about men, and also woman [have] their periods, and you know there might be some blood left over on the toilet and they didn't realize and it's embarrassing*. (A_MH_W_M_9)

Gender-specific staffing needs were also highlighted by staff and service users. Observations of need pertained to the therapeutic and care requirements of both men and women. Often service users expressed a wish for same gender staff:

*When I interacted with staff, it was mainly being like a male nurse who I had a rapport with much more so than with the female nurses. Not because I didn't like them, the female nurses, but with guys talk easier with guys - as simple as that*. (C_MH_M_M_3)

*I think they need to look hiring more women at the front for women, and maybe then, men for men .... My lady doctor, she gets it because she's a woman*. (C_MH_W_NM_5)

One staff member discussed the importance of having access to psychiatrists of both genders in the interests of provision of trauma-informed care.

*My personal preference is to have a male and a female psychiatrist on the ward … - but at the moment we've got two males in the ward - because some women, who … have had sexual abuse or trauma, don't like dealing with any of the men* (C_S_W_NM_01).

Another staff member pointed to the need to work with the individual and their preferences, rather than advocating for same gender staff to service user.

*Some male staff have a really good rapport with certain female patients. Some female patients can build a good rapport with male… It depends on the patient and the staff member*. (A_S_W_NM_3)

Gender was found to be a consideration in the staff-service user interactions, in terms of behavior and positioning of staff. This was seen in terms of protecting emotional and physical safety for the service user, but also to buffer staff from allegations of sexually inappropriate behavior or assault. Awareness of the possibility for false accusations of sexual assault by service users about staff was a concern for some male staff members:

*If you don't have a female to intervene, they could [make] accusations and say ‘this person did this to me'. And you, you know, you cannot say ‘no, no, no'. If they're all males, you know, it just puts our staff and also the patients… in a vulnerable situation*. (D_S_W_NM_1)

That noted, another staff member suggested that staff of both genders can be vulnerable to allegations of sexual inappropriateness or assault.

*the females aren't immune to [allegations of] rape as well. We get accused as well, you know … that's why I wish they'd make them segregated a bit better, because it just keeps them both safe*. (A_S_W_M_1)

Other concerns around staff safety were expressed by some staff including the need for staff to be careful when entering service user's bedrooms:

*If you're a female, and you've got a male client, and you're talking to that person in their bedroom… how safe are you?* (A_S_W_NM_3)

Several staff members explained that sometimes, in the interest of protecting staff, they would select a staff member of a particular gender to work with a service user depending on their presentation.

*One guy, he used to ring the nurses' bell, and then you'd go down there and he'd be masturbating. So when we get those type of guys, we get the male staff to check them*. (A_S_W_M_1)

**5. It goes beyond gender: Non-gender attributes that may influence risk and vulnerability on the unit**

Individual attributes, distinct from gender, were also described by some staff and service users as relevant to risk and vulnerability dynamics on the unit. These included those of age, physique, degree and type of mental unwellness, comorbidities including substance use, forensic history, trauma history, and power hierarchies that evolved through unit organization.

Age, for instance being younger or older than the majority of one's peers was mentioned several times by staff as a vulnerability, most often for women service users. Adult acute services in New Zealand accommodate people from 18 to 65 years of age. While youth and psychogeriatric facilities cater for ages outside of this bracket, staff noted that people eligible for these speciality services were occasionally admitted to general adult inpatient units, usually for short stays with “special” staff providing one-on-one vigil.

*Sometimes we get elderly patients and they have to be minded, or young, vulnerable females, they have to give them a minder* (A_S_M_NM_9)

Older people were sometimes observed to find the unit significantly anxiety provoking. Some of these observations connected perceived vulnerability to very real incidents of intimidation, coercion and bullying:

*For older people if they felt vulnerable, with somebody who might have been strongly coercing them to [give up] their food or their things and stuff, and they have to give it … [using] kind of stand-over tactics, and stuff … I have advocated the need to have a separate area for older service users* (D_S_W_NM_9)

Access to non-cash assets such as food and cigarettes, as well as unit liberties such as residence on the open unit were sometimes seen as impacting risk and vulnerability. Possession of cigarettes/tobacco was sometimes noted by staff as a risk due to its currency in the acute mental health setting where doors were locked restricting purchase and access to tobacco. Service users talked about coercion by other service users:

*It can be quite intimidating even with cigarettes … Other people taking your cigarettes off you. And if it's anybody too violent, and they want them, you just...I've handed all mine over before unwillingly. Cigarettes are a big problem in there actually … A lot of people have their leave, so they can just walk over [to the local shop]… so you definitely got access to go over the road, but if you've got a bully in there that wants them then … A guy bought a 50 gram [tobacco] the other day, and by the time everybody had hassled him, it was 50 grams of cigarettes in a day he provided everybody*. (A_MH_W_NM_8)

Service user forensic history was also a factor staff identified as impacting on vulnerability. A history of criminal offending was viewed by staff as having bearing on future risk of violent behaviors. Staff described service users who had prior involvement with the criminal justice system as “forensics” or as “forensic patients.” This suggests active categorization by staff that distinguishes these service users from their peers. Some staff comments pointed to a different expectation around behaviors, types of unwellness, and moral status of these individuals. Such comments were sometimes derogative in nature. Women and men were included in staff discussions of this risk.

*Like we've got one lady at the moment, she's ex-Forensics. And yeah, it's all … a risk. Because you've got to be aware of what's present and what's past* (B_S_W_NM_2)

*We were getting a lot of forensic patients here. And we're not supposed to say 'forensic patients'*. (A_S_W_M_1)

Different understandings of different mental illness diagnoses were also believed to impact on vulnerability, requiring different treatment or separation from others. For example, perception, albeit sometimes erroneous, of heightened risk of violence was regularly noted by staff and service users for some presentations:

‘*there's certain people that have been assaulted, myself included, where someone's really, really unwell. Paranoid, schizophrenic, organic'* (D_S_W_NM_2).

Other presentations, such as drug induced psychoses, as well as comorbid substance abuse issues, were also cited by staff as impacting on risk and vulnerability. A frequent notion of substance-related issues being different-in-kind from other forms of mental unwellness was articulated, with a different approach to treatment and assessment of risk sometimes proposed. One staff member described drug-related psychosis as a ‘completely different ball-game' to presentations of ‘true psychotic illness' (A_S_W_NM_2).

*We've had a few [people with methamphetamine-induced psychosis] come in, and it's quite scary to work with. You think, “Ooh” … They can be violent. They're disorganized, thought-disordered, driven, obviously. But yeah, is this the [right] place [for that]?* (B_S_W_NM_005)

Physical size and strength were also suggested as impacting risk or vulnerability status, in reference to both staff and service users. Generally, remarks suggested that bigger people, especially male service users, were perceived to be more dangerous, while smaller people, especially women, were perceived to be more vulnerable. One staff member commented, when discussing calming and restraint practices:

‘*it's going to come down to strength on strength … if you get a big guy' and ‘there's some big guys you're not going to be able to manage'* (A_S_M_M_7).

This said, there were some exceptions. A staff member noted that, although the bigger male staff might be commonly sent to deal with aggression on the unit, effective crisis management could involve women, and those of smaller physique:

‘*having said that, there is now a very small, petite, female [staff member] that comes over, and she's good'* (A_S_W_NM_3).

Further, a staff member noted that service user to staff violence does not follow a strict big-perpetrator-to-small-victim formulation:

*They don't want to hit the small female, but they've got no qualms hitting the big guy* (A_S_M_M_7)

In summary, clearly there are factors other than gender that impact on perceptions of risk and vulnerability on the unit, and there are likely to be complex interactions between these and the gendered factors reported by staff and service users in our study. Rather than a singular determinant of risk and vulnerability, gender was revealed to be a factor that could compound and have a multiplicative effect on other predictors of risk and vulnerability, including age, physical size, illness presentation, forensic history, psychosis and drug use.

## Discussion

The current heterogeneity of acute mental health facility structure and models of care suggests the question as to whether mental health units should be mixed-gender or gender segregated is moot. Proponents of both approaches tend to argue their position on grounds that include securement of emotional safety, physical safety, and the optimisation of on-unit therapeutic value in a gender vulnerabilities framework.

This paper began with the research question: “What are gendered experiences on the acute mental health unit, and what are the implications for facility design and models of care?”. In this framing of the issue our understanding of vulnerabilities in the acute mental health setting were, much like the literature, focused on gendered aspects of vulnerability. However, our findings suggest that while gender is a significant consideration in the delineation of vulnerability, the dynamic of vulnerability is more complex than we initially supposed. Interactions with and between gendered and non-gendered aspects of life on the mental health unit add to the impression of vulnerability.

We found that women service users spoke more frequently around trauma experiences, feelings of unsafeness, and hygiene differences, than their male service user counterparts. However, trauma preceding admission was not exclusive to women, and was often but not always characterized by gender-related events. Staff and service user perceptions around safety on the unit sometimes conveyed a male-on-female narrative of harm, but we found that some staff members viewed actual risk as genderless. We found some staff reported concerns around the accommodation of gender-diverse service users; some staff and a transgender service user, found unit organization and model of care inadequate to provide for the needs of these individuals. Staff and service users reported perceived gender differences in domains of recreation, nutrition, hygiene and staffing, although issues around hygiene and bathrooms were specific to units without ensuites, or units where ensuites were not the norm. The recreational and nutritional needs of male service users were seen in particular by staff, as inadequately accommodated. Women service users' concerns were most often aligned with trauma and fears about safety, whereas areas of concern for male service users were more often in relation to the objective fulfillment of activities of daily living.

However, non-gendered considerations with regards to vulnerability on the unit were also evident. Categories of vulnerability suggested by study participants include gender, but also age, forensic and trauma history, degree and kind of mental unwellness, substance use issues, physical size and strength, and access to currency including tobacco products and food.

Division of unit space that centers on gender difference demands substantiation of gender as a meaningful and effective distinction. Existing literature posits gender has real bearing on the emotional and physical safety of individuals working or resident on mental health units. Moreover, gender is seen as the delimiting factor by which care strategies, resources, and practical accommodations are directed to service user needs. This noted, ability of gender separation to mitigate gender-based violence and harassment on the mental health unit has been viewed with skepticism, due to the potential for this abuse to exist outside a heteronormative domain (3). Further, some literature does acknowledge a complexity that may exceed a gender-differences formulation (55). Our study highlights that gender is one of a number of interacting factors relevant to risk and vulnerability on the unit. Therefore, we advocate that those decisions around unit design and layout consider these factors in their multiplicity–with a mixed vulnerability lens. Furthermore, we argue that an exclusive focus on gender, or any other discrete factor, in the determination of unit layout is too reductive to meaningfully aid design.

### Implications for model of care and design

Our study uncovers a number of key findings, implications of which are that the architectural and built environment and model of care should consider a mixed vulnerabilities lens or framework. We suggest some key aspects of this below.

*A mixed-vulnerabilities framework* and *individualized care that considers identity and values the autonomy of service users – flexibility and choice-making*.

Assuming a mixed-vulnerabilities framework, the assessment of risk of violence and risk of being victimized is best rooted in an individualized, person-centered approach. Person-centered models of care promote strengths-based, flexible, identity-salient, and autonomy-promotive services, which can be mobilized through shared decision-making and self-directed support (79). A person-centered model of care, described as holistic, individualized, respectful and empowering (80), can benefit treatment engagement and treatment outcomes (58). This approach promotes service user decision-making in a context of, often, compulsory intervention where scope for choice is narrowed. Creation of choice-making opportunities on the unit can occur in the recruitment of gender diverse staff, in choices of meaningful recreation activities, in food choices, and in choices around treatment that may be formalized in an advance directive. Given shared identity or experience can create shared understanding, employing staff on the unit with diverse gender identities could provide service users more options in terms of with whom they build trust and rapport.

Our findings suggest that, while a situation of unmet needs and a paucity of options was described by many staff and service users, facilitation of individual choice-making does not require decisions informed by gender norms. For example, some service users and staff commented on a need for gender-suited activities–especially more physical activities and sports enjoyed by many males. Accommodating this need does not require a focus on gender differences. Providing a basketball hoop creates an opportunity for all who like shooting hoops to do so.

#### Trauma informed, trauma-sensitive care

Staff and service users acknowledged trauma as a precipitant and amplifier of mental unwellness. Some viewed this as a reason to segregate by gender. Service users have an increased risk of lifetime trauma, and markedly increased risk of having a post-traumatic stress disorder (PTSD) diagnosis compared with the general population (81). Women service users and staff most commonly referred to trauma histories in the unit population, recognizing the possibility of re-traumatisation in the unit environment. Seclusion, restraint, admission to the unit, and on-unit interactions with other service users were sometimes noted as traumatic events. Staff spoke of accommodations that could be made when working with individuals with trauma histories, including differences in managing restraint procedures, the importance of access to psychological therapies, and the use of “flexi” wings to insulate vulnerable service users or those more vulnerable in the stage of their unwellness, from the broader unit environment. Use of advance directives around restraint options, including seclusion; human restraint; and chemical restraint, would facilitate service user choice-making and add to ethos and practice of trauma informed care. We add to this set of recommendations the provision of ensuites to remove a further potential avenue for vulnerability. In Unit D, in which gender separation is largely absent, the provision of ensuites for every service user removed need for sharing of bathrooms.

Currently there is a mental health workforce shortage in New Zealand (82). The limited availability of on-unit psychological therapies and the inability of service users to have a say as to the gender of their clinicians indicate shortcomings in trauma-sensitive care. It should be noted that while trauma was mostly referenced in terms of women, trauma was not exclusive to women and separation by gender would not always be meaningful for trauma sensitive care. Our study finds need for individualized, rather than gender-categorized, accommodations of trauma.

#### Provision for safety, and clarity around safety-relevant protocols

Our study found that many service users and staff felt unsafe in the mental health unit. While participants sometimes implicated gender as relevant to risk, this view was not ubiquitous. Irrespective of gender's role in unit safety, measures to ensure the physical and emotional safety of people who reside and work on the unit are essential. Measures of surveillance, de-escalation, and practices and design features that assure privacy, are recommended and other recommendations to address safety on the unit have been explored in a related paper from this study (83). The units in our study had divergent approaches to these measures. Unit C used cameras to record activity in areas that had poor lines of sight. Unit D had more staff in the shared spaces (rather than situated in a nurse's station), which supported safety and could conceivably strengthen service user confidence on the unit. We recognize this as a progressive step for safety on the unit, more positive therapeutic relations and relational safety. More subtle forms of abuse, such as harassment and coercion are often less easy to monitor. Some staff spoke about safety-relevant protocols around sexuality and the initiation of romantic relationships between services users on the unit. Policies around intimate service user-service user relationships on the unit were noted as poorly defined, and some staff acknowledged a need for clearer and more proactive discussions around relationships that develop on the unit, service user sexual needs, and policies around service users having private visits from their significant others on the unit. Our study identifies need for more explicit discussion of unit policy around these issues with service user input.

#### Spaces in the built environment

The built environment can be designed in accordance with salutogenic principals (84) –comprehensibility, manageability and meaningfulness–to create an environment promotive of health. Often, however, units are conspicuously defensive–with architectures predicated on risk reduction, containment, lines of sight, and anti-ligature measures. This safety sometimes comes at the expense of autonomy and liberty (85). The Australasian Health Facility Guidelines lists consumer rights that include “the right to receive care in an environment with the least possible restriction” (86).

In two of the units studied (A and C), these corridors of bedrooms were gender-segregated. Shared spaces, such as courtyards and lounges, do seem to facilitate social interactions on the units. Service users and staff, however, often said these areas were too small, outdated, and noted a need for more flexibility within the shared spaces for privacy and social interaction. Overseas models of design, and recent research (87), suggests the use of booth seating (for privacy but inclusion) in communal spaces. The allocation of seating in lounge and dining areas is important to ensure service users do not feel excluded from shared spaces, and to promote choice-making in selection of where to sit. Provision of multiple communal spaces, including those allocated by gender, would facilitate safe interactions between service users, and would aid feelings of autonomy and privacy. Individuals would have choice in the determination of where to be, and who to be with.

Categorization of units as gender-segregated or gender-mixed is and continues to be problematic. Provision of gender segregated spaces within an otherwise mixed gender environment was common, with bathrooms, lounges and sleeping areas conferred to specific genders within a blended unit.

Incorporation of ensuites, advocated by the Australasian Health Infrastructure Alliance (88) has mitigated need for gender-specific bathrooms. Personal ensuites limit concerns around the mis-gendering of transgender and gender-diverse individuals. Provision of ‘designated lounges for special groups' based on perceived vulnerabilities including gender is a further recommendation. A statement from New Zealand's Ministry of Health (89) in 2002 on the design of inpatient facilities noted that ‘physical separation of male and female bedrooms and bathrooms now needs priority consideration for both privacy and safety reasons'. New unit design in New Zealand, such as that of Unit D in this study, heeds aspects of this guidance, with greater incorporation of “flexi” areas, ensuites, and the ability for service-users to use wristbands to lock, and to enter, and secure personal rooms.

In essence this study indicates that, with the help of creative architecture, gender becomes less relevant to overall unit design.

### Strengths and limitations

Our research draws from a qualitative study (74) that covers multiple case sites, and engages 85 interviewees, in a deep-dive of attitudes and perceptions around the aggregate of built, therapeutic and social elements that shape experience on the mental health unit. Our findings extend a small body of research that explores gendered experiences on acute mental health inpatient units. Semi-structured interviews with service users and staff promoted deep and organic discussion of gender on the unit. The service user voice is often missing in the literature around unit experience, and seldom sought from people who are residing on the units of the time of interviews. Although the four units selected as cases in this study had very different configurations, many of the themes around gender were consistent across settings. Interviews captured a breadth of staff and service user identities. Notably, staff inclusion was not limited to nursing staff, but also psychiatrists, cleaners, health care assistants, occupational therapists, consumer advisors, and kaimaanaki (Māori cultural workers). Existing qualitative literature provides an edifice for the understanding of problems inherent in the mixed and separational approaches to unit design and organization. Our study progresses this literature by moving beyond a *this or that* conceptualization of gender accommodations.

The exploration of gender on the unit was a corollary rather than the agenda of the broader study of mental health facility design from which data was sourced. This limited the amount of transcript data pertinent to gendered experience. Further, selection criteria governed by ethics considerations constrained service user participants to those who were deemed competent to provide informed consent. This meant the voices of those more acutely unwell service users were not captured. Acuity of illness, and differences of treatment, may bring a different set of gendered experiences. Those most unwell may be more likely to experience trauma on the unit–due to an increased risk of seclusion and restraint, and vulnerability related to unwellness or medication regimes. It should be noted, we did not ask specifically about staff violence toward service users, though questions were asked around whether service users felt safe.

Service user participants were interviewed while they were inpatients. While this avoids recall bias, some of the service users' perceptions may have been influenced by the symptoms they were experiencing, however this does not make any distress experienced less real. We deliberately did not ask about specific mental illnesses, symptoms or diagnoses as we did not want experiences explained away by particular conditions as it the common tendency and dominant medical discourse of much of the literature. We also note that past literature finds that service users are more likely to endorse or react favorably to the organization of the unit in which they are resident (41, 52). Further acquiescence biases and social acceptability biases may play into the interview process.

Finally, translatability across the 20 adult acute mental health facilities in New Zealand and other similar jurisdictions cannot be assumed. However, the diverse case study design and the consistency of many of our findings and narrative around gender and other aspect of vulnerability across these four cases will likely resonate with other researchers exploring the narratives of those with lived experience, residents and workers in acute mental health settings. Certainly, further research in similar overseas settings is warranted to test this out.

## Conclusion

Our study challenges the assumption that gender should be the central factor at play when considering unit design and layout strategies in acute mental health facilities. While we found that gender was a prominent aspect of participants' accounts of socialization, trauma, and violence on the unit, it is clear that other factors may be equally important in accommodation considerations. Our study located several co-variables that may have relevance to safety of unit staff and service users. While we find that no single identifier can be seen as sole motivator for unit demarcation, a multifactorial view of dynamics of risk and vulnerability could be used to guide unit organization, in a way where layout and social organization “flexes” with the needs on unit at any time. A person-centered approach–whereby assessment of needs, risks, vulnerabilities, and accommodations is tailored to the profile of an individual, is recommended by the study authors.

## Data availability statement

The datasets presented in this article are not readily available because in line with the ethics approval from HDEC ref (17/CEN/94), the full interview transcripts cannot be shared publicly. This is because of their highly sensitive nature and the personal accounts which make them potentially identifiable. For data inquiries please contact the University of Otago Human Ethics Committee through the following webpage: https://www.otago.ac.nz/council/committees/committees/HumanEthicsCommittees.html.

## Ethics statement

The studies involving human participants were reviewed and approved by the Central Health and Disability Ethics Committee (17/CEN/94). The patients/participants provided their written informed consent to participate in this study.

## Author contributions

Funding acquisition, ethics and design of study, and data collection was undertaken by GJ. Identification of themes, data analysis, and writing original draft was led by EM with all authors contributing to reviewing and editing. Triangulation of themes in the data analysis was undertaken by all authors. All authors contributed to the article and approved the submitted version.

## Funding

This work was supported by a Marsden Fast Start from the Royal Society of New Zealand under Grant UOO1623.

## Conflict of interest

The authors declare that the research was conducted in the absence of any commercial or financial relationships that could be construed as a potential conflict of interest.

## Publisher's note

All claims expressed in this article are solely those of the authors and do not necessarily represent those of their affiliated organizations, or those of the publisher, the editors and the reviewers. Any product that may be evaluated in this article, or claim that may be made by its manufacturer, is not guaranteed or endorsed by the publisher.
